# Overcoming Barriers to Lung Cancer Screening by Implementing a Single-Visit Patient Experience

**DOI:** 10.15436/2377-0902.17.1469

**Published:** 2017-05-17

**Authors:** Cherie P. Erkmen, Ryan F. Moore, Clifford Belden, Verdi DiSesa, Larry R. Kaiser, Grace X. Ma, Anuradha Paranjape

**Affiliations:** 1 Department of Thoracic Medicine and Surgery, Lewis Katz School of Medicine, Temple University, Philadelphia, PA; 2 Center for Asian Health, Lewis Katz School of Medicine, Temple University, Philadelphia, PA; 3 Department of Surgery, Temple University Hospital, Philadelphia, PA; 4 Department of Radiology, Lewis Katz School of Medicine, Temple University, Philadelphia, PA; 5 Department of Clinical Sciences, Lewis Katz School of Medicine, Temple University, Philadelphia, PA; 6 Department of Medicine, Lewis Katz School of Medicine, Temple University, Philadelphia, PA

## Introduction

In 2011 the authors of the National Lung Screening Trial (NLST) reported a 20% risk reduction of lung cancer death using annual screening with a low dose CT (LDCT)^[1]^. In 2013, the United States Preventative Services Task Force (USPSTF) gave lung cancer screening a grade B recommendation indicating that LDCT should be covered by private insurers without cost sharing (co-pay)^[2]^. Implementation of a screening program could potentially avert 12,000 deaths annually^[3]^. Unfortunately, implementation of lung cancer screening programs has been slower than expected. Despite evidence that USPSTF recommendations influence 88.4% of practicing primary care providers (PCP’s)^[4]^, only 47% of PCPs acknowledged the lung cancer screening recommendation^[5]^, and only 12% of PCPs in an academic setting used LDCT scan for lung cancer screening^[6]^. The reasons for the failure to generate enthusiasm to implement lung cancer screening are multifactorial. However, lung cancer screening provides an opportunity to design novel patient-centered care. In this discussion, we will explore both the barriers to lung cancer screening implementation and our multidisciplinary approach which prioritizes patient-centered care by creating a single-visit patient experience.

### Barriers to Lung Cancer Screening Implementation

Introduction of a new screening protocol presents a challenge in any clinical practice. Major barriers include educating health care providers on current evidence regarding lung cancer screening, implementing shared decision making (SDM) and creating a sustainable infrastructure with mechanisms to ensure quality care.

#### Applying Current Evidence to Patient Care

The American Academy of Family Physicians (AAFP) released a statement on lung cancer screening concluding that the evidence is insufficient to recommend for or against it^[7]^. The AAFP cited that the USPSTF recommendation was based largely on NLST which enrolled healthy volunteers. An argument exists that results of the NLST may not be generalizable in that 90 percent of those enrolled were Caucasian, 59% were male and 31.5% were college educated^[8]^. Furthermore, lung cancer screening introduces the possibility of potential harm from false positive results. Ninety five percent of the radiographic positive studies ultimately were shown to be false positive^[9]^, with 2.5% of these receiving an invasive procedure and 0.3% experiencing a major complication. False positive results also induce some level of stress and anxiety on the part of the patient and time and effort for follow up on the part of the health care provider. Providers considering lung cancer screening must evaluate the current evidence, assess applicability to the individual patient and balance the potential risks and benefits. Before recommending screening, providers have to be sufficiently convinced of the overall benefits.

#### Shared Decision Making (SDM)

SDM is an essential component of lung cancer screening according to the authors of NLST, CMS recommendations, USPSTF guidelines, and professional societies^[1,2,10]^. Specifically, SDM is a process whereby a provider and the patient identify a health issue, recognize the options that exist, discuss potential harms and benefits of these options, incorporate patient values and preferences, and explicitly choose and enact a plan^[11]^. Unfortunately, there are no available guidelines for SDM in lung cancer screening though decision aids such as should iscreen.com^[12]^ and optiongrid.org^[13]^ can guide discussions. Specific recommendations regarding which provider should participate in SDM, the timing of the discussion and the depth of content to review are lacking. This paucity of guidelines often results in a failure of SDM implementation. Despite NLST study data published in 2011, in 2012 fewer than 20% of patients eligible for lung cancer screening engaged in a discussion potential benefits and harms of screening with LDCT. This number further decreased after the release of the USPSTF guidelines in 2014^[14]^. If SDM is a critical part of lung cancer screening and providers do not have sufficient support to conduct SDM, they may opt out of recommending lung cancer screening altogether.

#### Infrastructure

Lung cancer screening requires an infrastructure in order to be implemented successfully. Providers must develop a mechanism of identifying eligible patients within their practice. Simply eliciting an accurate smoking history can be challenging and time-consuming within the confines of a busy practice. Additional time is accrued as providers must determine whether a patient is covered for lung cancer screening. Those covered though Medicare or commercial insurance should be eligible under the CMS coverage statement and the Affordable Care Act, respectively. However, providers ordering lung cancer screening must gain approval from each patient’s insurance, a process that can vary in time and effort. Providers must also prepare a protocol for addressing patients without insurance or those whose policy does not cover screening. CMS guidelines mandate explicit requirements of documentation, notes accompanying a SDM visit and a written order with specific information regarding a patient’s eligibility. Patients then must be referred to an approved imaging facility that submits data to a CMS-approved registry and employs qualified radiologists to interpret the LDCT and they must have a reliable mechanism for following up on screening results and communicating the results to patients.

Providers wishing to routinely implement lung cancer screening must also build a coordinated smoking cessation infrastructure. The discussion regarding lung cancer risk, a component of the screening protocol, provides an excellent opportunity to address smoking cessation in active smokers. Smoking cessation as a component of lung cancer screening may confer an additional benefit to patients by potentially reducing the risk of other smoking-related disorders. However, coordination of lung cancer screening and smoking cessation requires resources and time.

Finally providers must have access to multidisciplinary teams for evaluation and treatment of findings that accrue from lung cancer screening. In a given medical practice, there must be a sufficient number of patients to justify the infrastructure required, a factor somewhat of a challenge given that only 7 – 17% of the United States population between the ages of 55 and 80^[15]^ are eligible for lung cancer screening.

#### Financial and Legal Responsibility

The cost of lung cancer screening and the question as to who pays underscores the fact that reimbursement for screening remains under evaluation. A cost effective analysis within the NLST revealed that each quality adjusted life year (QALY) gained would cost $81,000^[16]^, well below the accepted $100,000 threshold for value within healthcare^[17]^. However, in clinical practice, the health care provider arranging for lung cancer screening must incur the costs relating to patient recruitment, implementing SDM, arranging for payment, directing patients to follow up care and following up with reimbursement deficiencies. Though CMS covers SDM visits, the payment may be insufficient to cover the actual investment of time and money. Health care providers also assume a legal responsibility. In ordering a lung cancer screening study, the provider assumes responsibility for following upon positive screens, incidental findings and yearly scans for eligible patients. Failure of adequate follow up could result most importantly in harm to patients but also expose the provider to legal action. Likewise, failure to screen an otherwise eligible patient could be a potential source of a legal claim. Some health care providers may not have the ability to address all of the financial and legal implications of lung cancer screening, and therefore refrain from referring eligible patients.

### Overcoming Barriers to Provide Patient-Centered Lung Cancer Screening

Lung cancer screening is a potentially life-saving intervention when implemented with evidence-based protocols, SDM and a reliable infrastructure. Unfortunately, many individual providers have limited resources to fully implement lung cancer screening in this way. Institution-based, multidisciplinary screening programs have the advantage of significant resources to support and coordinate all of the providers needed for lung cancer screening. Sharing the responsibility of lung cancer screening among providers within a comprehensive program improves patient-centered care.

At Temple University Hospital we developed a comprehensive lung cancer screening program focused on the patient experience. Our lung cancer screening program coordinates care into a single visit for the patient. Our program consists of a clinical team of primary care physicians, pulmonologists, SDM specialists, radiologists, medical and radiation oncologists, thoracic surgeons, interventional radiologists, smoking cessation specialists and pharmacists. We have the ability to assess each patient’s eligibility, arrange for coverage of the SDM visit and the LDCT, utilize decision aides for SDM, perform and interpret LDCTs, discuss results with patients, arrange for appropriate follow up care and counsel on smoking cessation when applicable, all on the same day. Our team provides these services by aligning the expertise of our clinicians with the diverse responsibilities of screening. Our program hopes to decrease patient’s anxiety and worry by consolidating counseling before and after the LDCT into a single patient visit at a single location.

The diverse responsibility of screening does not rely on a single person or specialty. Instead, we assign specific roles and responsibilities to team members. With critical responsibilities, we built a redundancy into our screening protocol. For instance, we created a redundancy of reporting LDCT scan results to patients. Our radiologists document LDCT scan results using the American College of Radiology reporting system, lung-RADs, in the patient’s electronic medical record on the same day as the scan. This report is accessible to the patient and his/her health care provider. The lung cancer screening specialist seeing the patient also interprets the LDCT, confirms concordance with the radiologist’s interpretation, and discusses the results with the patient on the same day as the LDCT. If there is a concerning finding, the radiologist will contact the lung cancer screening specialist to ensure accurate reporting to the patient. When appropriate, our program initiates referral to a pulmonary specialist, thoracic surgeon or other indicated specialty services. Our approach of shared responsibility among multiple disciplines decreases the chance of failed action on a screening result.

Our lung cancer screening program partners with our surrounding community to support lung cancer screening. To communicate emerging evidence about lung cancer screening to our referring providers, we developed several educational programs including continuing medical education (CME) events, visits with health care providers in their offices and a curriculum for residency programs. We have created and shared templates for lung cancer screening orders and SDM notes. These templates guide providers through documentation of CMS-required elements including age, smoking history and other lung cancer screening inclusion criteria. Details of lung cancer screening evidence and specifics of our program are available in printed and web-based form to providers. Health care providers referring patients to our program may choose which services they would like for their patients. For example, a physician in our community may provide an SDM experience or refer patients to SDM specialists within our lung cancer screening program. Providers may counsel patients on smoking cessation, but still refer eligible patients for lung cancer screening and smoking cessation counseling to further influence smoking behavior. Referring providers know that we can accomplish any or all services in a single patient visit and communicate the result to patients and their health care providers in a timely fashion.

Over the past 14 months, we have screened 258 eligible patients under this single-day protocol. Of the 258 patients, 128 (49.61%) were male, 130 (50.39%) were female, 160 were African American (62%), 69 were Caucasian (26.7%), 25 were Hispanic (9.7%), and 4 were Asian (1.6%). Research measuring patient satisfaction, anxiety and decision regret under this screening protocol is underway. Our program incorporates bioinformatics specialists, epidemiologists and a research infrastructure to collect demographic and outcomes data for further study of lung cancer screening. With our integrated research infrastructure, we are able to communicate our regional lung cancer screening experience to providers. These relevant data give providers the confidence to refer patients who were underrepresented in NLST for lung cancer screening.

## Conclusion

In conclusion, implementation of lung cancer screening is time and resource intensive. Few individual health care practitioners have the ability to assume all of the responsibilities of lung cancer screening including staying abreast of emerging data, implementing evidence-based protocols including SDM and maintaining an infrastructure with mechanisms to ensure quality care. We propose a new paradigm, namely a coordinated effort between health care providers and a multidisciplinary lung cancer screening team that prioritizes patient-centered care. Future directions for our group include further study into this paradigm and its sustainability.

## References

[1] Aberle DR, Adams AM, Berg CD (2011). Reduced lung-cancer mortality with low-dose computed tomographic screening. N Engl J Med.

[2] Moyer VA (2014). Screening for lung cancer: U.S. Preventive Services Task Force recommendation statement. Ann Intern Med.

[3] Ma J, Ward EM, Smith R (2013). Annual number of lung cancer deaths potentially avertable by screening in the United States. Cancer.

[4] Lewis JA, Petty WJ, Tooze JA (2015). Dose CT lung Cancer Screening Practices and Attitudes among Primary Care Providers at an Academic Medical Center. Cancer Epidemiol Biomarkers Prev.

[5] Raz DJ, Wu GX, Consunji M (2016). Perceptions and Utilization of Lung Cancer Screening Among Primary Care Physicians. J Thorac Oncol.

[6] Klabunde CN, Marcus PM, Silvestri GA (2010). primary care physicians’ lung cancer screening beliefs and recommendations. Am J Prev Med.

[7] (2013). Clinical Preventive Service Recommendation, Lung Cancer.

[8] Seehusen DA (2014). Should family physicians routinely screen for lung cancer in high-risk populations? No: The USPSTF’s recommendation for lung cancer screening is overreaching. Am Fam Physician.

[9] Leventhal W (2012). Primary Care Perspective on Lung Cancer Screening. J Thoracic Imaging.

[10] Jaklitsch MT, Jacobson FL, Austin JH (2012). The American Association for Thoracic Surgery guidelines for lung cancer screening using low-dose computed tomography scans for lung cancer survivors and other high-risk groups. J Thorac Cardiovasc Surg.

[11] Makoul G, Clayman ML (2006). An integrative model of shared decision making in medical encounters. Patient Educ Couns.

[12] (2013). Lung cancer CT screening. Should I get screened?.

[13] (2017). WA health Care Authority. Option Grid decision aids.

[14] Carter-Harris L, Tan AS, Salloum RG (2016). Patient-provider discussions about lung cancer screening pre- and post-guidelines: Health Information National Trends Survey (HINTS). Patient Educ Couns.

[15] U.S. Preventive Services Task Force (2013). Final Recommendation Statement Lung Cancer: Screening.

[16] Black WC, Gareen IF, Soneji SS (2014). Cost-Effectiveness of CT Screening in the National Lung Screening Trial. N Engl J Med.

[17] Neumann PJ, Cohen JT, Weinstein MC (2014). Updating cost-effectiveness -the curious resilience of the $50,000-per-QALY threshold. N Engl J Med.

